# Ovulatory-cycle frozen embryo transfer: spontaneous or triggered ovulation and the impact of LH elevation at hCG triggering

**DOI:** 10.1038/s41598-023-34017-x

**Published:** 2023-05-03

**Authors:** Asaf Bilgory, Yuval Atzmon, Nardin Aslih, Yasmin Shibli Abu Raya, Moamina Sharqawi, Maya Shavit, Daniela Estrada, Einat Shalom-Paz

**Affiliations:** 1 IVF Unit, Department of Obstetrics and Gynecology, Hillel Yaffe Medical Center, Hadera, Israel; 2 Present Address: Ruth and Bruce Rappaport School of Medicine, The Technion Institute of Technology, Haifa, Israel

**Keywords:** Health care, Medical research

## Abstract

The effect of the luteinizing hormone (LH) elevation before the human chorionic gonadotropin (hCG) trigger in ovulatory frozen-thawed embryo transfer (Ovu-FET) cycles has not been determined. We aimed to investigate whether triggering ovulation in Ovu-FET cycles affects the live birth rate (LBR), and the contribution of elevated LH at the time of hCG trigger**.** This retrospective study included Ovu-FET cycles performed in our center from August 2016 to April 2021. Modified Ovu-FET (hCG trigger) and true Ovu-FET (without hCG trigger) were compared. The modified group was divided according to whether hCG was administered, before or after LH increased to > 15 IU/L and was twice the baseline value. The modified (n = 100) and true (n = 246) Ovu-FET groups and both subgroups of the modified Ovu-FET, those who were triggered before (n = 67) or after (n = 33) LH elevation, had comparable characteristics at baseline. Comparison of true vs. modified Ovu-FET outcomes revealed similar LBR (35.4% vs. 32.0%; P = 0.62), respectively. LBR were similar between the modified Ovu-FET subgroups regardless of the hCG trigger timing (31.3% before vs. 33.3% after LH elevation; P = 0.84). In conclusion, LBR of Ovu-FET were not affected by hCG trigger or whether LH was elevated at the time of hCG trigger. These results add reassurance regarding hCG triggering even after LH elevation.

## Introduction

Cryopreservation technologies have developed dramatically and vitrification has replaced slow freezing methods, allowing improved post-warming survival rates^1^ and increased tendency to freeze embryos^2^.

The indications for freezing embryos are diverse and include fertility preservation, preventing ovarian hyperstimulation syndrome, and preimplantation genetic testing, among others^3^. Approaches to frozen-thawed embryo transfer (FET) include natural cycle (NC), letrozole ovulation induction cycle, or hormone replacement therapy (HRT) cycle^4,5^. Each approach has its advantages and disadvantages. With ovulatory FET (Ovu-FET) cycle, which includes natural and letrozole-induced ovulatory cycles, a more physiological ovarian function is used, as the corpus luteum secretes endogenous hormones. Compared with HRT cycles, Ovu-FET is associated with lower risk of hypertensive disorders of pregnancy^6,7^. Hormonal blood levels do not need to be monitored in HRT cycles; therefore, fewer clinic visits are required and these can be adjusted according to the patient’s and physician’s schedules. Overall, the ideal FET cycle has not yet been determined^5,8^.

Ovu-FET involves close monitoring of follicular growth with hormonal blood samples and transvaginal ultrasound scans. The monitoring is intended to detect the timing of the luteinizing hormone (LH) surge and the subsequent progesterone (P4) elevation, which determines the timing of FET. Ovu-FET can be further divided into modified Ovu-FET, in which human chorionic gonadotropin (hCG) is given to trigger ovulation in order to synchronize the FET. In true Ovu-FET, ovulation is not triggered by hCG. The rationale for triggering is to increase the accuracy of ovulation timing and therefore, enable scheduling the FET for a more convenient time that suits both the fertility unit and the patient. One example is to avoid cycle cancellation when FET should be performed according to ovulation and the age of the embryo, on a day the unit is closed. Another advantage of modified Ovu-FET is that hCG is given before ovulation is documented, potentially reducing the costs of extra visits for hormonal blood monitoring and repeated ultrasound scans^9^.

While a recent, randomized, controlled trial showed that clinical pregnancy rates did not differ based on whether hCG was given^10^, Fatemi et al. suggested NC without hCG was better than with hCG^11^. In their study, a prespecified, interim analysis found a significantly higher ongoing pregnancy rate of 31.1% in the true NC-FET group, compared to 14.3% in the modified NC-FET group, and the study was terminated early. This finding raised a concern regarding the timing and the necessity of the hCG trigger, particularly considering the hypothesis that hCG and LH share the same receptor and their simultaneous presence could affect pregnancy adversely. Weismann et al. expressed their concerns regarding this randomized controlled trial, claiming several biases^12^. The main issue was that women were triggered by hCG when they were already ovulating. It is important to emphasize that Fatemi et al. reported that the hCG trigger was given when the mean LH level was 17.5 ± 16.7 IU/L and P4 was 0.91 ± 1.6 ng/mL. These high LH and P4 values might reflect scenarios in which ovulation has already occurred and triggering with hCG might adversely affect outcomes.

The effect of the LH elevation before the hCG trigger has not been determined. To the best of our knowledge, even though several studies have compared modified and true FET, most did not evaluate LH and P4 values on the day of the hCG trigger^10,13–15^.

The aim of this retrospective study was to investigate the live birth rate (LBR) in Ovu-FET with or without hCG trigger, and to assess whether LH elevation before hCG trigger adversely affected the LBR in Ovu-FET.

## Methods

### Patients

This retrospective cohort study was conducted in a single IVF department. Records of all patients and their cryopreserved embryos from cycles performed August 2016 through April 2021 were analysed. Data collected included maternal age at freezing and FET, body mass index (kg/m^2^), gravidity, parity, infertility diagnosis, and ovarian reserve parameters at the time of stimulation cycles, such as baseline follicular stimulating hormone and antral follicle count. FET cycle parameters included the stage of embryo development (cleavage or blastocyst), number of embryos transferred, embryo morphokinetic scoring by time-lapse imaging, embryo morphology scores, use of letrozole for stimulating dominant follicle growth, use of hCG to trigger ovulation, P4 for luteal support, endometrial thickness (mm) measured with transvaginal ultrasonography on the day of ovulation or hCG trigger day, and the use of 0.1 mg triptorelin (Decapeptyl®, Ferring, Caesarea, Israel) 7 days after ovulation day for luteal support.

To reflect the broad range of patients typically encountered in clinical practice, only a few exclusion criteria were applied regarding baseline characteristics. These included women with Mullerian uterine anomalies, and donor egg and surrogacy cycles. This research was exploratory in nature; therefore, we chose not to use strict inclusion and exclusion criteria to avoid limiting the potential scope of the study. Only cycles in which transfers were performed were included. Each cycle was counted separately, with some women having more than one cycle.

### Embryo quality assessment

Embryo quality was assessed for cleavage and blastocyst stage according to the standard classifications^16–18^. Top-quality cleavage stage embryos included the following parameters: > 4 equal-size blastomeres on day 2 or > 6 equal-size blastomeres on day 3, ≤ 20% fragmentation and no multinucleate cells. Top-quality blastocysts included excellent and good day 5/6 embryos. Morphological grades of blastocysts were assessed, as follows: excellent (3–6 AA), good (3–6 AB, 3–6 BA), average (3–6 BB), or poor (3–6 BC, 3–6 CB, 3–6 CC)^19^. Embryo morphokinetic scoring using time-lapse imaging according to known implantation data scores (KIDS) and European Society of Human Reproduction and Embryology (ESHRE) consensus were available for about half of the transferred embryos^18,20^.

### Ovu-FET protocols

Growth of the dominant follicle was based on a natural cycle or induced by letrozole. In letrozole-induced ovulatory cycles, women received 5 mg letrozole (Teva, Netanya, Israel) daily for 5 days, from day 5 to day 9 of the menstrual cycle. When the leading follicle was ≥ 17 mm, and endometrial thickness was ≥ 7 mm, as seen on transvaginal ultrasound, ovulation was triggered with 250 mcg of recombinant hCG (Ovitrelle^®^; Merck-Serono, Darmstadt, Germany) or was anticipated to occur naturally. The default was to not trigger ovulation. The decision to trigger ovulation was based mainly on the need to schedule the transfer to avoid weekends and holidays and to shorten the follow-up. The timing of the hCG trigger was determined according to LH and P4 blood levels, dominant follicle diameter and endometrial thickness. Day ‘0’ was set to start 36 h after administration of hCG. LH was considered elevated at the time of hCG triggering if it was > 15 IU/L and twice the baseline value measured at the early follicular stage (days 2 to 5 of the cycle). hCG was not given if the P4 level was already > 1 ng/mL. In cases when the LH surge was anticipated to occur naturally, day ‘0’ was determined to be the day estradiol levels started to decline and P4 began to increase above 1.5 ng/mL.

Luteal phase support commenced on the FET day. It included oral dydrogesterone 10 mg TID (Duphaston^®^ Abbott, Chicago, IL, USA), vaginal micronized P4 inserts 100 mg TID (Endometrin^®^, Ferring, Caesarea, Israel), 8% vaginal micronized P4 gel 90 mg BID (Crinone^®^, Merck Serono, Darmstadt, Germany), or intramuscular P4 100 mg daily (Prontogest^®^, Raz Pharmaceutics, Kadimah, Israel).

### Pregnancy determination

Treatment and pregnancy outcomes were monitored until delivery. Chemical pregnancy was defined when serum hCG level was > 25 mIU/ml 12–14 days after FET but failed before it could be confirmed clinically by ultrasonography. Clinical pregnancy was confirmed when a gestational sac with a fetal heartbeat was visible on ultrasound exam, at 6–7 weeks of gestation. Extrauterine pregnancy was diagnosed if an extrauterine gestational sac was evident or whenever serum hCG level was > 1500 mIU/L, without evidence of intrauterine gestational sac.

### Main outcome measures

The primary outcome was the LBR, defined as the number of deliveries that resulted in at least one live-born neonate after 24 weeks of gestation divided by the number of FET cycles. The secondary outcomes were the chemical pregnancy and miscarriage rates. The chemical pregnancy rate was defined as the number of chemical pregnancies divided by the number of FET cycles. The miscarriage rate was defined as the number of clinical pregnancy losses divided by the number of FET cycles.

### Statistical analysis

Statistical analysis was performed using SPSS (IBM Corp., New York, NY, USA). NC and letrozole-induced ovulatory cycles were grouped as Ovu-FET. Ovu-FET were first divided into cycles with or without hCG trigger. The sample size was calculated using OpenEpi, version 3^21^. Assuming a power of 80%, 95% confidence interval, and differences in LBR between the two groups of 20%, at least 100 women were needed in each group. Afterwards, a sub-analysis of the cycles with hCG trigger was conducted according to whether the hCG was administered before or after LH elevation. We used the Shapiro–Wilk test to evaluate the data distribution. When appropriate, comparisons were analysed using Student’s t-test or Mann–Whitney U test. Proportions were compared using Chi-Square or Fisher exact test. P-values < 0.05 were considered significant.

### Ethics approval

The study was approved by the Institutional Review Board of Hillel Yaffe Medical Center (approval number 0026-20-HYMC, February 2020). All procedures were conducted in accordance with the ethical standards of the institutional research committee and with the 1964 Declaration of Helsinki and its later amendments or comparable ethical standards.

### Consent

Patient consent was waived due to the retrospective nature of the study.

## Results

A total of 346 ovulatory FET cycles were included. These included 100 modified Ovu-FET (with hCG trigger) and 246 true Ovu-FET (without hCG trigger). Table 1 presents the demographic data and baseline characteristics. There were no significant differences between groups.

**Table 1:** Demographic data and baseline characteristics of modified vs. true Ovu-FET cycles.

Variable	Modified Ovu-FET; n = 100	True Ovu-FET; n = 246	p-value
Woman’s age at freezing, years^a^	32.9 ± 6.5	32.6 ± 6.4	0.63
Woman’s age at FET^a^	33.8 ± 6.5	33.6 ± 6.2	0.81
Body mass index (kg/m^2^)^a^	26.1 ± 5.6	24.9 ± 4.9	0.06
Gravidity^a^	1.24 ± 1.15	1.35 ± 1.59	0.53
Parity = 0, n (%)	45 (45%)	135 (54.9%)	0.098
Parity ≥ 1, n (%)	55 (55%)	111 (45.1%)	
Infertility diagnosis, n (%)			0.94
Mild-moderate male factor	18 (18%)	44 (17.9%)	
Severe male factor	30 (30%)	79 (32.1%)	
Mechanical	19 (19%)	39 (15.9%)	
Unexplained	30 (30%)	79 (32.1%)	
Polycystic ovary syndrome	3 (3%)	5 (2.0%)	
Follicle-stimulating hormone (IU/L)^a^	6.7 ± 2.4	7.0 ± 2.4	0.24
Antral follicle count^a^	21.85 ± 10.0	21.28 ± 10.3	0.64

*FET* frozen embryo transfer, *Ovu-FET* ovulatory cycle FET.

^a^Mean ± SD.

Table 2 presents the FET cycle characteristics and outcomes. There were no significant differences in embryo parameters, including number of embryos transferred, developmental stage, or quality of embryos based on morphology and morphokinetics. The cycle parameters were comparable, including endometrial thickness on the day of hCG trigger or LH surge, use of letrozole for stimulation of dominant follicle growth and decapeptyl for luteal support.

**Table 2:** Cycle characteristics and outcomes of modified vs. true Ovu-FET cycles.

Characteristic	Modified Ovu-FET; n = 100	True Ovu-FET; n = 246	p-value
No. frozen embryos transferred	1.26 ± 0.46	1.25 ± 0.45	0.88
1	75 (75%)	186 (75.6%)	
2	24 (24%)	58 (23.6%)	
3	1 (1%)	2 (0.8%)	
Embryo transfer			0.22
Cleavage-stage	61 (61%)	147 (59.8%)	
Blastocyst	35 (35%)	96 (39.0%)	
One cleavage-stage + one blastocyst	4 (4%)	3 (1.2%)	
TOP quality embryos among total number of embryos (%)	111/125 (88.8%)	263/306 (86.0%)	0.53
Mean KIDS^a^	4.66 ± 0.84 (n = 51)	4.46 ± 0.95 (n = 166)	0.17
Mean Alpha ESHRE^a^	2.21 ± 0.64 (n = 51)	2.15 ± 0.85 (n = 166)	0.59
Endometrial thickness (mm)^a^	8.99 ± 1.84	9.41 ± 2.16	0.094
Decapeptyl for luteal support	90 (90%)	235 (95.5%)	0.079
Letrozole induced ovulation cycle	22 (22%)	72 (29.3%)	0.06
Progesterone for luteal support			
No luteal support	0 (0%)	3 (1.2%)	0.3
Endometrin	40 (39%)	55 (22.4%)	0.013
Crinone	5 (5%)	20 (8.1%)	0.36
Duphaston	53 (53%)	162 (65.9%)	0.028
Prontogest	2 (2%)	6 (2.4%)	1.00
Outcome per embryo transfer cycle			
No pregnancy	59 (59%)	129 (52.4%)	0.28
Chemical pregnancy rate	4 (4%)	5 (2.0%)	0.29
Miscarriage rate	5 (5%)	25 (10.2%)^b^	0.14
Live birth rate	32 (32%)	87 (35.4%)	0.62

*ESHRE* European Society of Human Reproduction and Embryology, *KIDS* known implantation data score, *Ovu-FET* ovulatory cycle frozen embryo transfer.

^a^Mean ± SD.

^b^Two extrauterine pregnancies were included in this group.

Medications used for luteal support in the modified Ovu-FET cycle compared to true Ovu-FET differed. Endometrin was used more often in the modified Ovu-FET (39.0% vs. 22.4%, P = 0.013) cycles and Duphaston was used more often in true Ovu-FET (65.9% vs. 53%, P = 0.028). LBR were comparable (32.0% in the modified Ovu-FET group vs. 35.4% in the true Ovu-FET group; P = 0.62). Mode of delivery, whether normal vaginal delivery, vacuum-assisted, or Cesarean delivery, was comparable between groups. Chemical pregnancy and miscarriage rates were comparable (Table 2).

Tables 3 and 4 present the sub-analyses of patients triggered with hCG, according to the hCG trigger before or after LH elevation. Table 3 presents the demographic data and baseline characteristics comparing 67 cycles in which hCG was given before LH elevation and 33 cycles in which hCG was given after LH increased > 15 IU/L and its baseline value doubled.

**Table 3:** Demographic data and baseline characteristics in modified Ovu-FET cycles according to hCG triggering before vs. after LH elevation.

Characteristic	hCG before LH elevation; n = 67	hCG after LH elevation; n = 33	p-value
Age at freezing^a^	33.5 ± 6.3	31.8 ± 6.8	0.24
Age at FET^a^	34.4 ± 6.4	32.5 ± 6.6	0.17
Body mass index (kg/m^2^)^a^	26.0 ± 5.4	26.2 ± 6.1	0.85
Gravidity^a^	1.18 ± 1.01	1.36 ± 1.38	0.45
Parity = 0, n (%)	27 (40.3%)	18 (54.5%)	0.20
Parity ≥ 1, n (%)	40 (59.7%)	15 (45.5%)	
Infertility diagnosis, n (%)			0.19
Mild-moderate male factor	13 (19.4%)	5 (15.2%)	
Severe male factor	17 (25.4%)	13 (39.4%)	
Mechanical	15 (22.4%)	4 (12.1%)	
Unexplained	19 (28.3%)	11 (33.3%)	
Polycystic ovary syndrome	3 (4.5%)	0 (0%)	
Follicle-stimulating hormone (IU/L)^a^	6.47 ± 2.3	7.09 ± 2.6	0.23
Antral follicle count^a^	20.88 ± 9.4	23.8 ± 11.1	0.17

*FET* frozen embryo transfer, *LH* luteinizing hormone, *Ovu-FET* ovulatory cycle FET.

^a^Mean ± SD.

**Table 4:** Characteristics and outcomes of modified Ovu-FET cycles according to hCG triggering before vs. after LH elevation.

Characteristic	hCG before LH elevation; n = 67	hCG after LH elevation; n = 33	p-value
Baseline LH level (early follicular, IU/L)^a^	8.1 ± 3.3	6.8 ± 2.7	0.075
LH level on the day of hCG administration (IU/L)^a^	10.9 ± 4.3	31.8 ± 13.0	< 0.001
P4 level on the day of hCG administration (ng/mL)^a^	0.29 ± 0.19	0.50 ± 0.29	< 0.001
No. of frozen embryos transferred^a^	1.30 ± 0.49	1.18 ± 0.39	0.24
1	48 (71.6%)	27 (81.8%)	
2	18 (26.9%)	6 (18.2%)	0.47
3	1 (1.5%)	0 (0%)	
Embryo transfer, n (%)			0.24
Only cleavage-stage	38 (56.7%)	23 (69.7%)	
Only blastocyst	25 (37.3%)	10 (30.3%)	
One cleavage-stage + one blastocyst	4 (6.0%)	0 (0%)	
TOP quality embryos among total number of embryos (%)	78/86 (90.7%)	31/39 (79.5%)	0.09
Mean KIDS^a^	4.6 ± 0.87 (n = 35)	4.63 ± 0.80 (n = 16)	0.9
Mean Alpha ESHRE^a^	2.2 ± 0.72 (n = 35)	2.31 ± 0.48 (n = 16)	0.58
Endometrial thickness (mm)^a^	8.94 ± 1.66	9.10 ± 2.18	0.69
Decapeptyl for luteal support	62 (92.5%)	28 (84.8%)	0.29
Letrozole-induced ovulation cycle	17 (25.4%)	5 (15.2%)	0.2
Progesterone for luteal support			0.54
Endometrin	29 (43.3%)	11 (33.3%)	
Crinone	3 (4.5%)	2 (6.1%)	
Duphaston	33 (49.2%)	20 (60.6%)	
Prontogest	2 (3.0%)	0 (0%)	
Outcome per embryo transfer cycle			
No pregnancy	40 (59.7%)	19 (57.6%)	0.84
Chemical pregnancy rate	3 (4.5%)	1 (3.0%)	0.73
Miscarriage rate	3 (4.5%)	2 (6.1%)	0.73
Live birth rate	21 (31.3%)	11 (33.3%)	0.84

*ESHRE* European Society of Human Reproduction and Embryology, *hCG *human chorionic gonadotropin, *KIDS* known implantation data score, *LH* luteinizing hormone, *Ovu-FET* ovulatory cycle frozen embryo transfer.

^a^Mean ± SD.

Table 4 shows the characteristics and outcomes of the cycles. Except for LH and P4 levels on the day of hCG administration, all other baseline demographic and FET cycle characteristics were similar between the groups. The LBR were comparable, with 31.3% in those who received the hCG before the spontaneous LH elevation vs. 33.3% after it(P = 0.84). The mode of delivery was comparable between groups, as were secondary outcomes.

## Discussion

This study evaluated outcomes after modified vs. true ovulation in Ovu-FET protocols. Similar to other studies, we demonstrated that modified and true Ovu-FET yielded comparable results^10,13–15^. As we further focused on the group of modified Ovu-FET, we compared cycles in which hCG was given before or after the onset of the LH surge. We found similar characteristics in both groups and comparable outcomes, regardless of LH elevation.

The impact of elevated LH at the time of the hCG trigger in FET cycles is an unresolved issue. LH acts primarily in gonadal theca cells, but extragonadal activity is present, as well. Indeed, the expression of LH receptors in the human endometrium is evident, according to microarray analyses and reverse transcriptase-polymerase chain reaction studies^22^. The kinetics of expression of LH/hCG receptors in the endometrium suggests that these receptors could even constitute a marker of endometrial receptivity^23^. The delicate interactions between the embryo and the endometrium are crucial for timing the window of implantation. They are affected by several factors, including the hormonal milieu^24^. If these interactions are considered, it can be assumed that triggering with hCG after LH has already started to increase could be problematic because the endometrium was already exposed and activated by LH. Therefore, the window of implantation could theoretically shift and cause implantation failure due to asynchronous endometrium.

In our IVF unit, modified Ovu-FET is usually triggered with hCG before the LH surge. However, in some scenarios, the FET day was scheduled by triggering with hCG after the LH had already started to rise. For example, when LH was starting to rise but P4 levels were still low, waiting for spontaneous ovulation might require transfer on a weekend when the unit is closed. The hCG trigger led to scheduling the FET a day later in these cases. We believe that although theoretically, triggering with hCG when LH was elevated can lead to asynchronous endometrium and poorer outcomes, in practice the effect is apparently minor when LH has not peaked and P4 levels are < 1 ng/ml.

Several studies have compared true and modified FET and the results reported here support those findings. In a retrospective study, Groenewoud et al. reported similar ongoing pregnancy rate^25^. They compared 122 modified NC-FET in which hCG was given after LH surge to 111 modified NC-FET in which hCG was given before LH surge. However, they defined the LH surge as > 10 IU/L. This level seems very low and may reflect baseline LH levels because they were not reported in their study, which adds to the uncertainty regarding their conclusions. Our results support those of an interesting retrospective study that encouraged even greater flexibility regarding timing of the hCG trigger^26^ It compared 1,163 modified NC-FET according to different categories of LH levels on the day of hCG. Group B (n = 245) had LH 15–24.9 IU/L; group C (n = 253) had LH 25–39.9 IU/L and group D (n = 383) had LH ≥ 40 IU/L. They added a group A that included women who lived far from the hospital and for whom next-day clinic attendance was not practical. Therefore, their last LH was assessed the day before the hCG day, at a mean of 14.8 ± 15.4 IU/L. They found that outcomes were not significantly different among the groups and concluded that hCG could be administered any time between the start of the LH surge (LH > 15) and up to peak LH levels (LH > 40), without harming clinical outcome, The current study defined elevated LH as twice the baseline and > 15 IU/L. Importantly, hCG was not administered when the P4 level was > 1 ng/mL, as this reflects post-ovulatory levels. Our protocol reflects pre-ovulatory timing without exposure to P4, which might be a reason for the significantly lower ongoing pregnancy rate in the hCG group of Fatemi et al., with a P4 of 0.91 ± 1.6 ng/mL^11^.

In the current study, the only difference between the modified and true groups was the luteal phase support. It was a coincidence that women who received Endometrin for luteal support were more likely to undergo modified Ovu-FET, while women who received Duphaston were more likely to undergo true Ovu-FET, as luteal support was determined at the discretion of the physician in charge. Nonetheless, according to the ESHRE 2019 guidelines for ovarian stimulation for IVF/ICSI, no major differences in efficacy were found when comparing the different administration routes of P4^27^. Moreover, studies comparing micronized P4 and Duphaston, both in fresh and in FET cycles, demonstrated comparable outcomes^28–30^.

The main limitation of the current study was its retrospective design, with the potential resulting biases. The study was not powered to detect differences in the sub-analysis, which constitutes a significant limitation. Another limitation is derived from the definition of the onset of the LH surge. There is no consensus in the literature on the exact parameters that define the LH surge and the actual ovulation day in Ovu-FET^31^. The current study defined it as when LH was above 15 IU/L and had doubled from its baseline value in the early follicular phase. Ovulation day was defined as when the estradiol levels were starting to decline and P4 levels were > 1.5 ng/mL. Another aspect we did not address was the cost-effectiveness of the treatment. In NC, the follow-up criteria that include ultrasound scans and hormonal blood monitoring increase costs. However, our center is government supported and compensation is per FET cycle, regardless of the number of follow-up visits. Moreover, more scans may increase the cost per transfer, but cycle cancelation is even more expensive.

The strengths of this study include that it involved a large cohort from one center during a relatively short period in which all embryos were frozen by vitrification. Taken together, these factors decrease the potential differences in practice. In addition, accurate baseline LH levels were reported, as well as on the day of hCG trigger.

In conclusion, this study contributes information that helps clarify inconsistencies regarding triggering ovulation with hCG in Ovu-FET cycles, particularly after the LH surge has begun. The outcomes of the Ovu-FET cycles were comparable regardless of whether ovulation was triggered before or after the LH surge had begun. These results seem reassuring and indicate that hCG can be given even after the LH surge has started, if P4 is < 1 ng/mL. We believe that our findings present a flexible approach, which allows hCG triggering of ovulation after the LH surge had started, without compromising FET outcomes. This strategy allows greater flexibility in scheduling the FET day. By administering hCG, the hormonal changes that determine the window of implantation can be adjusted in a way that can suit the schedules of the patients and the clinic, with potentially fewer cancelations due to weekend and holiday closures, and without any detrimental effects on the desired outcome. Well-planned trials with larger patient populations, including cost-effectiveness assessments, are needed to develop and guide best practices.

## Data Availability

Data will be made available upon reasonable request to the corresponding author.

## Abbreviations

ESHRE: European Society of Human Reproduction and Embryology
FET: Frozen-thawed embryo transfer
hCG: Human chorionic gonadotropin
KIDS: Known implantation data scores
LBR: Live birth rate
LH: Luteinizing hormone
NC: Natural cycle
Ovu-FET: Ovulatory frozen-thawed embryo transfer
P4: Progesterone
